# Heightened sensitivity in high-grooming honey bees (Hymenoptera: Apidae)

**DOI:** 10.1093/jisesa/ieae057

**Published:** 2024-05-28

**Authors:** Derek Micholson, Robert W Currie

**Affiliations:** Department of Entomology, University of Manitoba, 218-12 Dafoe Road, Winnipeg, Manitoba R3T 2N2, Canada; Department of Entomology, University of Manitoba, 218-12 Dafoe Road, Winnipeg, Manitoba R3T 2N2, Canada

**Keywords:** grooming behavior, *Varroa* mite resistance, sensitivity, stimulus

## Abstract

Honey bees use grooming to defend against the devastating parasite *Varroa destructor* Anderson and Trueman. We observed the grooming responses of individual bees from colonies previously chosen for high- and low-grooming behavior using a combination of mite mortality and mite damage. Our aim was to gain insight into specific aspects of grooming behavior to compare if high-grooming bees could discriminate between a standardized stimulus (chalk dust) and a stimulus of live *Varroa* mites and if bees from high-grooming colonies had greater sensitivity across different body regions than bees from low-grooming colonies. We hypothesized that individuals from high-grooming colonies would be more sensitive to both stimuli than bees from low-grooming colonies across different body regions and that bees would have a greater response to *Varroa* than a standardized irritant (chalk dust). Individuals from high-grooming colonies responded with longer bouts of intense grooming when either stimulus was applied to the head or thorax, compared to sham-stimulated controls, while bees from low-grooming colonies showed no differences between stimulated and sham-stimulated bees. Further, high-grooming bees from colonies with high mite damage exhibited greater grooming to *Varroa* than high-grooming colonies with only moderate mite damage rates. This study provides new insights into *Varroa*-specific aspects of grooming, showing that although a standardized stimulus (chalk dust) may be used to assess general grooming ability in individual bee grooming assays, it does not capture the same range of responses as a stimulus of *Varroa*. Thus, continuing to use *Varroa* mites in grooming assays should help select colonies with more precise sensitivity to *Varroa*.

## Introduction

The parasitic mite *Varroa destructor* is considered the single greatest threat to apiculture worldwide (Rosenkranz et al. 2010), and even low infestations of the mite can have significant economic impacts (Currie and Gatien 2006). With the exception of Africanized honey bee populations and some lines of bees bred for *Varroa* resistance, most honey bee colonies not treated for *Varroa* mites will usually perish within 2–3 years (Korpela et al. 1992, De Jong 1996). Since honey bees are valuable pollinators that provide critical pollination services to around one-third of crops worldwide (McGregor 1976, Delaplane et al. 2000, Klein et al. 2007), understanding the mechanisms of resistance against parasites and diseases is of critical importance.

One natural defense against *Varroa* is grooming behavior, a form of social immunity where bees use their legs and mandibles to remove mites from themselves (auto-grooming) or from nestmates in the colony (allo-grooming) (Peng et al. 1987), reviewed by Pritchard (2016). Allo-grooming may be solicited through the grooming invitation dance (Haydak 1945, Land and Seeley 2004). Grooming behavior has previously been shown to be able to restrain the growth of *V. destructor*, particularly in colonies of the Asian honey bee, *Apis cerana* Fabricius, and to a lesser degree in colonies of the Western honey bee, *Apis mellifera* Linnaeus (Peng et al. 1987, Buchler et al. 1992, Russo et al. 2020). Further studies have found higher levels of grooming across different subspecies of *A. mellifera*, including Africanized bees (descendants of *A. mellifera scutellata*) (Aumeier 2001, Guzman-Novoa et al. 2012, Invernizzi et al. 2015, Nganso et al. 2017), bees from the Primorsky Krai region of Russia (Rinderer et al. 2001, Guzman-Novoa et al. 2012), and *Apis mellifera carnica* (Ruttner and Hänel 1992, Thakur et al. 1997). Grooming behavior has also been found to be a key component of tracheal mite (*Acarapis woodi*) resistance in *A. mellifera* (Pettis and Pankiw 1998) and serves a general function in the removal of pollen, dust, or other irritants that bees regularly encounter when foraging (Winston 1987).

Enhanced grooming is a heritable trait (Moretto et al. 1993, Stanimirovic et al. 2010, Arechavaleta-Velasco et al. 2012, Hamiduzzaman et al. 2017) of interest to breeding programs for *Varroa*-resistance behaviors. However, many methods of assessing grooming behavior are indirect and labor-intensive. Usually, they involve collecting and counting all mites falling onto sticky papers placed beneath hives and calculating the mite mortality rate (Currie and Tahmasbi 2008) or microscopic identification of physical damage to collected mites and calculating the percentage of damaged mites (Guzman-Novoa et al. 2012, Hunt et al. 2016). In addition, both methods are imperfect because overall mite fall may be attributed to other *Varroa*-resistance behaviors, such as *Varroa*-sensitive hygiene (Rinderer et al. 2010), and damage to fallen mites may also occur as a result of other factors unrelated to the direct effects of grooming such as natural mortality (Boecking and Spivak 1999).

Observing the grooming responses of individual bees after stimulation with *Varroa* mites, or an alternate stimulus, is another method of analyzing grooming behavior (Aumeier 2001, Land and Seeley 2004, Guzman-Novoa et al. 2012, Rivera-Marchand et al. 2012, Bąk and Invernizzi et al. 2015, Bąk and Wilde 2015, Hamiduzzaman et al. 2017, Dadoun et al. 2020, Morfin et al. 2020a, 2023, Russo et al. 2022) and these studies provide direct evidence of the behavior of individual bees taken from source colonies. For example, small particles of chalk dust applied to the wing base have previously been used to stimulate grooming responses in *A. mellifera* held in observation hives (Land and Seeley 2004). In other experiments, various aspects of grooming behavior have been characterized after adding live *Varroa* mites onto bees in Petri dish “arenas” (Aumeier 2001, Guzman-Novoa et al. 2012, Bąk and Wilde 2015) and colonies that have been selected for resistance to *Varroa* have also demonstrated greater grooming responses compared to susceptible colonies in these types of experiments (Guzman-Novoa et al. 2012, Rivera-Marchand et al. 2012, Invernizzi et al. 2015, Dadoun et al. 2020, Russo et al. 2022). Guzman-Novoa et al. (2012), for example, linked the intensity of grooming responses and the successful removal of mites from individual bees with the proportion of damaged mites (PDM) found on source colony sticky papers as well as the overall colony mite infestation level, providing additional evidence for using these direct methods of observation in addition to or in place of indirect methods. The age of bees selected for assays is also important. Dadoun et al. (2020) and Russo et al. (2022) showed young nurse bees engage in more grooming responses than older workers aged 14 days (Russo et al. 2022) and 21 days (Dadoun et al. 2020).

The mechanisms of grooming behavior that result in individual bees being able to detect and remove irritants or mites from their bodies remain poorly understood but undoubtedly involve multiple steps. For example, to auto-groom, an individual worker must first recognize a stimulus and then respond in a generalized or specific way to remove the irritant (or mite). Hamiduzzaman et al. (2017) suggested that high-grooming bees may have heightened abilities to perceive stimuli in their environment either through tactile or chemosensory means. Morfin et al. (2020a) looked at stimulating the grooming responses of presumed *Varroa*-resistant Africanized bees and presumed *Varroa*-susceptible European (Carniolan) bees with either *Varroa* or an alternate irritant of 20 mg of wheat flour placed onto the thorax, and found that Africanized bees responded significantly faster than European bees when stimulated with either *Varroa* or flour. Thus, they concluded that irritants other than *Varroa* might be used to differentiate between genotypes of bees with different levels of grooming behavior and have used wheat flour trials in subsequent grooming studies (Morfin et al. 2023).

However, further research is needed to verify the sensitivity of high-grooming bees to *Varroa* versus alternate stimuli. For example, bees that are effective groomers may have heightened overall sensitivity to any stimulus and exhibit generalized responses, while bees that groom and bite *Varroa* may have more sophisticated sensory capabilities that may not be discerned in bioassays using standardized irritants. It also remains unclear what the sensitivities of different body regions are in bees that have enhanced grooming abilities. Thus, researching the differences in these sensitivities and abilities is important to better understand how bees defend against *Varroa* and how *Varroa* might evade grooming. If grooming is a polygenic trait that requires a combination of genes to be successful, as some suggest (Stanimirovic et al. 2010, Arechavaleto-Velasco et al. 2012, Hamiduzzaman et al. 2017, Morfin et al. 2020b, 2023), then breaking down the different aspects of this complex behavior will provide information to improve assays that can then be used for the development of phenotypic and molecular markers.

The overall goal of this research was to focus on whether individual bees from high-grooming colonies are better able to respond to mites or other irritants through enhanced grooming and to assess if bees from high-grooming colonies had greater sensitivity than low-grooming bees to a standardized stimulus administered to different body regions. An experiment was designed to simultaneously test 3 separate hypotheses: (i) Bees from colonies chosen for exhibiting high-grooming behavior (using a combination of 2 selection metrics) have heightened sensitivity to a stimulus from irritants (both *Varroa* as well as a standardized chalk dust stimulus (calibrated to be equivalent to the weight of a mite) relative to colonies characterized as low-grooming; (ii) Individual bees from colonies that exhibited high-grooming behavior at the colony level will be more sensitive to a stimulus of *Varroa* than to both a sham treatment with a short touch and a standardized but longer-exposure irritant of equivalent mass to a *Varroa* mite (chalk dust); and (iii) Bees from high-grooming colonies have enhanced sensitivity to a standardized stimulus over different body regions compared to low-grooming bees.

## Materials and Methods

### Selection of Colonies

The experiment was carried out at the University of Manitoba in Winnipeg, MB, Canada (49°54ʹ N, 97°14ʹ W). High- and low-grooming colonies for use in laboratory assays were selected in the spring of 2017 based upon an assessment of mite drop (DMMR and PDM) from broodless units from a total of 200 colonies that were part of the Genome Canada Bee’Omics project at the University of Manitoba in 2016. Colonies used in that experiment originated from a variety of sources, including donations from 9 different producers throughout the province of Manitoba (*n* = 83), diverse local stock from the University of Manitoba (*n* = 75), and imported packages from New Zealand (*n* = 42). In the spring of 2016, the queens from each parent colony were first caged, and broodless colonies were established by shaking bees from the respective colonies into sealed cardboard hive boxes. Worker populations were standardized at 1 kg of bees, equivalent to approximately 8,000 bees. Caged queens were placed into the hive boxes along with the associated bees from their own colonies, and the new broodless colonies were held overnight under cool conditions in total darkness in the University of Manitoba’s overwintering building before being moved to their new locations. To complete the setup of the larger experiment, 200 broodless units were then randomly separated and transferred to 4 separate apiaries the following morning to emulate commercial-sized bee yards and minimize risks associated with loss of yards. Frames of bees were transferred out of the sealed cardboard hive boxes into standard Langstroth 10-frame brood chambers. Each hive was then immediately outfitted with a commercially available “*Varroa*-nator” screened bottom board (Dimo’s Tool & Die, 12 Bangor Ave, Winnipeg, MB, Canada), below which were placed Vaseline-type sticky boards (made from freezer paper) (Calderone and Lin 2003) to measure mite fall over 2 consecutive 72-h periods. Then, to increase acceptance of the queens to their new environments, the queens were slow-released from their cages by removing the corks from the cage entrance and plugging it instead with a mixture of wax and honey to allow the bees to slowly chew the entrance open. Ninety-eight percentage of queens were accepted to begin the BeeOmics experiments in 2016, and the 4 hives where queens were not accepted were removed from the experiment. In addition, any hives with queens that swarmed or superseded during the experiments were excluded from further testing to avoid testing progeny of new queens (i.e., new genetics).

*Varroa* populations for each broodless unit were standardized to the extent possible. The initial mean abundance of mites (mites per 100 bees) was measured by means of an alcohol wash from the parent colony by averaging 2 samples of roughly 250 bees each. The target range for the initial infestation of colonies was 0.5–2.0 mites per 100 bees. No colonies used in the 2016 Manitoba Bee’Omics experiment exceeded the 2% maximum infestation at the establishment; however, colonies that had an initial infestation of less than 0.5% were inoculated with live *Varroa* mites collected from highly infested colonies (kept in a separate mite-rearing yard at the University of Manitoba) using a modified CO_2_ shake method that has proven to be effective in establishing mite populations in cage trials and full-size colonies (Ebadi et al. 1980, Currie and Tahmasbi 2008, Human 2013). On the day of colony establishment, mites were collected to inoculate those colonies needing additional mites. Forty-five mites were placed into Petri dishes prepared with round pieces of moistened paper towel, which were then stored in Styrofoam coolers containing Ziploc bags filled with warm tap water (roughly 30 °C). Colonies needing additional mites were inoculated as quickly as possible the same day (i.e., within 2 h of mite collection). The paper towels containing the mites were placed under the colony lids and onto the top bars of frames to allow the mites to crawl into the hives and onto the bees (Ostermann and Currie 2004, Currie and Tahmasbi 2008, Bahreini and Currie 2015). Any mites remaining in the dishes were transferred to the paper using a fine-tipped paintbrush. The total number of mites present in each starting broodless colony was then estimated by taking the % infestation from the parent colony samples multiplied by the estimated total number of bees (8,000) and adding 45 mites to that if extra inoculation occurred.

The daily mite mortality rate (DMMR) (i.e., the proportion of total mites falling to bottom boards each day) was then assessed for each colony by means of collecting the 2 (72 h) rounds of sticky papers from the colonies. During this time, any mite fall would have been attributed either to natural mite death or the grooming behavior of bees since no brood (of a stage that *Varroa* would infest) was yet present. All mites that dropped onto sticky papers were carefully removed in the lab, counted, and kept in vials containing 70% ethanol until they were later analyzed for signs of damage. The DMMR for each colony was calculated by taking the total number of mites that dropped from each colony over the 6 days (144 h) divided by the estimated total number of mites in each colony at the establishment, divided by 6.

Each collected mite was viewed under a dissection microscope at 40× magnification for signs of damage to legs, mouthparts, or ventral shields (mites with dented idiosoma were not categorized as damaged). The level of damage was further classified as mild, moderate, or heavy. Mild damage was when mites had parts of 1 leg missing or partial damage to mouthparts or ventral shield; moderate damage was when mites had a whole leg or whole mouthparts missing; and heavy damage was when mites had multiple legs missing or had multiple damages (Andino and Hunt 2011, Hunt et al. 2016) (see Supplemental Material for images of damaged mites). The PDM for each colony was calculated by dividing the total number of mites with *any* signs of damage by the total number of mites collected off sticky papers. PDM calculations and rankings for the high-grooming group were based on a cutoff of at least 5 mites analyzed, and of those colonies selected, the minimum 6-day mite drop was 7 mites.

**Fig. 1.**
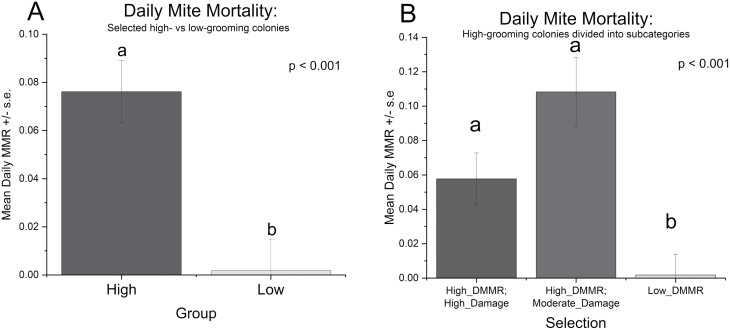
Mean DMMR ±  SE for A) high- and low-grooming groups of colonies selected based upon DMMR, and B) high- and low-grooming groups of colonies with the high-grooming colonies further divided into high- and moderate-damage subcategories. Means followed by the same letter are not significantly different (*P* < 0.05, Tukey).

A combination of the DMMR and the PDM was then used to choose presumed high-grooming colonies for subsequent experiments; however, only the DMMR was used to choose low-grooming colonies (see Fig. 1). DMMRs for the selected high-grooming colonies were all considered high but were further subdivided based on damage rates as described below. Of the selected high-grooming colonies, DMMRs ranged from 0.026 to 0.17 (proportion of total mites dropping per day), and PDMs ranged from 0.17 to 0.71 (proportion of collected mites with any damage). The PDM values were subdivided into moderate and high subcategories: damage rates between 0.17 and 0.30 were categorized as moderate, and those above 0.30 were categorized as high—which was based on an equivalent to the reported damage rates in previous studies (Andino and Hunt 2011, Guzman-Novoa et al. 2012, Hunt et al. 2016, Russo et al. 2020, 2022).

Prior to any laboratory assays in 2017, the colonies were overwintered indoors in the University of Manitoba Bee Lab’s wintering building over the 2016–2017 winter at a temperature of 5 °C and held under constant darkness with standard ventilation (Gruszka and Currie 1998). All colonies used in assays were treated with 3 rounds of 65% liquid formic acid in Spring 2017 to equalize *Varroa* infestation levels at or close to 0% prior to performing bioassays.

Eleven high- and 11 low-grooming colonies were chosen for testing and were moved on 14 June 2017 to an apiary at “The Point” at the University of Manitoba, positioned at least 50 m away from any other colonies. Colonies were arranged randomly in a row spaced roughly 1 m apart, and entrances were angled to discourage the drifting of bees between colonies. A variety of different colored boxes were also used for hive bodies to provide orientation cues and further discourage drifting. Colonies were managed throughout the season in an attempt to prevent swarming and queen supersedure by clipping queen cells and providing extra space and ventilation as required. In cases where colonies eventually swarmed or superseded, they were assayed before the population turned over to avoid testing progeny from the new queen (i.e., new genes).

### Individual Grooming Behavior Trials

Individual bee trials began on 20 June 2017 and ran until 18 August 2017. Two colonies were tested at a time (one high-grooming and one low-grooming) to control for any seasonal variation in grooming. Each morning, about 100 bees from a brood frame (capped or open) from each colony being tested were collected and split into two 150 ml collection vials (i.e., ~50 bees per vial) with one screened end and a sliding lid that allowed for individual bees to be removed one at a time. A sugar cube was placed into each collection vial as a temporary food source for the duration of the trials. Live *Varroa* mites were collected each morning using the same CO_2_ shaking method referenced above. About 30 mites were collected each morning and divided into 2 Petri dishes containing around 15 mites each. A moist paper towel was placed in each dish to prevent desiccation of mites, as well as 4–5 worker pupae that had been removed from a brood frame that morning to serve as a food source for the duration of the trial. Petri dishes with mites were again kept warm by placing them into a small Styrofoam cooler with a Ziploc bag full of warm water (~30 °C) and were brought to the lab along with the bees.

Experimental trials took place in the flight room in the honey bee research laboratory (Pernal and Currie 2002). The flight room was maintained at a constant temperature of 25 °C (similar to temperatures in Aumeier 2001 and Morfin et al. 2020a) for the duration of the trials with full spectrum lighting in the room. To begin each trial, 5 bees were transferred from the collection vial into a 50-ml Falcon tube by opening the sliding lid and letting them crawl out. To assist with applying the stimulus treatments, the bees were then briefly anesthetized with CO_2_ by means of a small hose attached to the lid of the Falcon tube, which should minimize any impact of CO_2_ on bee behavior (Ebadi et al. 1980, Human 2013). A flow meter was used to control the flow of CO_2_ to 2 L/min, and the gas was turned on for a count of 8 s, which provided just enough CO_2_ to gently anesthetize the bees for the time needed to transfer them to Petri dishes and apply treatments. The 5 bees were transferred into 5 new Petri dishes on a table outfitted with a white bench liner which provided contrast to help analyze the behavior of the bees. Treatments were then randomly assigned to each dish using 5 plastic discs, each representing 1 of the 5 treatments. Once the bees were in the dishes, a video camera (iPhone 5 attached to a ring clamp stand positioned above the dishes) was started that captured video of all 5 dishes in the trial. The treatments were then administered to the bees when they were beginning to move around again. In cases where the bees did not move soon after the anesthetization, the bee was replaced with another (following the same procedure).

The 5 treatments were: (i) *Control*: a soft touch with a small paintbrush (Heinz Jordan white Taklon series 970 size 10/0) to the thorax; (ii) *Mite*: one live *Varroa* mite placed onto the bee (usually the thorax) using a small paintbrush; (iii) *Head*: a puff of chalk dust applied to the head; (iv) *Thorax*: a puff of chalk dust applied to the thorax; and (v) *Abdomen*: a puff of chalk dust applied to the abdomen. For all chalk dust treatments, Mastercraft marking chalk was used and was administered via a Gilson P100 adjustable pipette with a disposable tip loaded with chalk dust. The pipette had been calibrated so that one depression of the plunger released approximately 0.3 mg of chalk dust, which we found to be roughly equal to the weight of one mite after weighing multiple *Varroa* mites with a CAHN 25 automatic electrobalance (Model 5725). After the stimuli were applied, the dishes were covered with modified Petri dish lids that had the plastic top cut out and replaced with Mylar film with about 20–30 holes punched in it with a small nail to allow for airflow (lids were cleaned after each trial with 70% ethanol and reused for subsequent trials; however, new Petri dish bottoms were used for each trial).

Immediately after the last stimulus application, a 3-min timer was started, and when the time was up, the video recording was stopped, and the trial was ended. Since 2 colonies were being tested on the same day, trials for each colony were alternated to avoid any effects of the bees sitting in the room waiting to be tested (e.g., trial #1 for colony 1, followed by trial #1 for colony 2, trial #2 for colony 1, trial #2 for colony 2, and so on). Twenty-five trials (consisting of each of the 5 different treatments) were completed for each colony. This usually took 2 or (rarely) 3 days to complete; however, new bees and mites were used each day. At the end of each day, videos were saved to be analyzed later for grooming responses. In total, 2,750 bees were tested (25 worker bees were tested in each of the 5 treatments for each of the 11 colonies in each of the high- and low-grooming groups). The colony was used as the experimental unit.

### Video Analysis

We quantified the following components of grooming behavior: time to start grooming, time spent on light grooming, time spent on intense grooming, total grooming time (light + intense grooming), and the number of grooming “bouts.” Light and intense grooming responses were categorized using the same methods as Guzman-Novoa et al. (2012)—i.e., light grooming consisted of slower swipes with just 1 or 2 legs at most, and intense grooming consisted of more vigorous swiping using a minimum of 2 legs. Any mite removals were also recorded, and if so, the bee was watched for an additional 1 min to record whether the bee interacted with the mite in the dish. With that data, we calculated the proportion of time spent on light grooming, the proportion of time spent on intense grooming, the total proportion of time spent grooming, and the average length of each grooming “bout.”

Video clips for each trial were analyzed using PowerDVD 16 Media Player, which allowed the video observer to adjust both the zoom and playback speed to accurately observe and quantify the grooming responses of each individual bee. Thus, video clips for each trial were watched 5 times, once for each bee in each stimulus treatment.

Three observers watched and analyzed the video to collect data. However, to minimize subjectivity, observers were trained carefully, and care was taken to calibrate each observer’s observations by analyzing 25 bees together before each observer analyzed recordings independently. Observers knew the colony numbers for the videos they analyzed; however, observers were kept blind to which group the colony was in (e.g., high- or low-grooming) to avoid biasing the results.

Grooming responses were quantified using an iPhone and a customized grooming timer “app” programmed by Josh Usiskin (*dev.usiskin.ca:51947/#!/trial*). For each bee, the observer would start the grooming timer to coincide with the point at which the stimulus was applied to the bee in the video, or in cases where the bee had not yet fully recovered from the effect of the CO_2_, the grooming timer was started when the bee was standing on all legs and appeared to have recovered from the anesthetic. On rare occasions where bees were assessed as not having recovered from the anesthetic during video analyses, these were noted as such, and data were not used for statistical analyses. The grooming timer was designed such that it ran for 3 min, and the observer could time how long the bee groomed itself by holding 1 finger down on the screen for the full duration of any light grooming instances or 2 fingers for instances of intense grooming. Thus, if during one bout of grooming, the bee switched between light and intense grooming, the observer could also easily switch between 1 and 2 fingers. When the 3-min grooming timer ended, grooming times were displayed in a spreadsheet in the app.

### Statistical Analysis

For colony selection, the DMMRs and PDM were analyzed using a one-way analysis of variance (ANOVA) to compare chosen groups of putative high- and low-grooming colonies, as described in Section 2.1 (PROC MIXED, SAS Institute Inc. 9.4, 2016). Residuals of data were first plotted visually in R (Version 1.3.1056) to examine normality and homogeneity of variance. Since the data were nonnormal, an arcsine square root transformation (Arcsine (Squareroot (*X*))*57.3) was applied to the data. Where *F*-tests indicated differences were observed between selected groups, a post hoc Tukey’s test was used to compare differences among the 3 groups. For bioassay experiments, the proportion of time spent on intense grooming was chosen as the most appropriate metric to compare among selected groups as intense grooming has previously been identified as one of the most important grooming metrics and one that is more closely associated with successful mite removals in individual bee assays (Guzman-Novoa et al. 2012, Hamiduzzaman et al. 2017). To analyze the proportion of time spent on intense grooming, a split-split-plot ANOVA design was used in which the colony was considered the experimental unit and the selection method was a main plot factor, treatment was a subplot factor, and trials were the sub-sub plot. Colony (selection-method), colony*treatment (selection-method), and trial*colony (selection-method) were random effects. Prior to analysis, residuals were plotted visually in R (Version 1.3.1056) to check normality and homogeneity of variance; however, statistical analyses were performed in SAS (SAS Institute Inc. 9.4, 2016). Since data were not normal, an arcsine square root transformation was performed as described above. All data are presented as untransformed means. A variance components covariance structure was selected based on the lowest Akaike information criterion, and the degrees of freedom were calculated using the ddfm = Kenward Rodger option to adjust for poor homogeneity of variance. Interactions between the selection-method and treatment were partitioned using the SLICE option in the LSMEANS statement, and individual contrasts were used to compare treatment means within each selection group (protected LSD, PDiff PROC MIXED, SAS Institute Inc. 9.4, 2016). Data for other metrics that were quantified can be found in the University of Manitoba’s Dataverse: https://dataverse.lib.umanitoba.ca/dataset.xhtml?persistentId=doi%3A10.34990%2FFK2%2FGSOPG6.

## Results

### Colony Selection

**Fig. 2.**
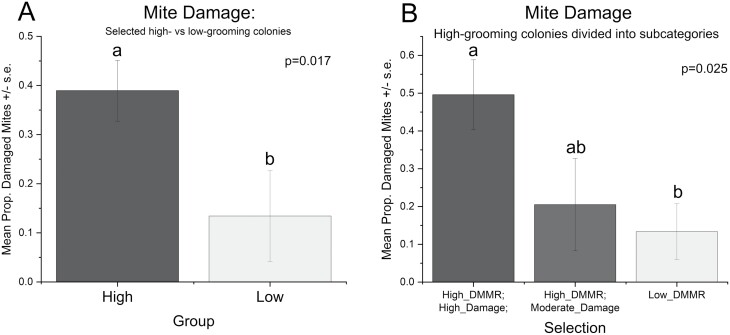
Mean PDM ± SE for A) high- and low-grooming groups of colonies selected based on DMMR and B) high- and low-grooming groups of colonies with the high-grooming colonies further divided into high- and moderate-damage subcategories. Means represented by bars followed by the same letter are not significantly different (*P* < 0.05, Tukey).

Mean DMMR was significantly higher in the pool of high-grooming colonies selected based on high-DMMR than in the low-grooming colonies (*F*_1,20_ = 43.18, *P* < 0.001) (Fig. 1A). When the high-grooming colonies were further subdivided into high- and moderate-damage subgroups (Fig. 1B), there also was a significant effect of the selection method (*F*_2,19_ = 27.60, *P* < 0.001) on DMMR. DMMRs for both the high-DMMR/high-damage subgroup (*P* = 0.0001, Tukey) and the high-DMMR/moderate-damage subgroup (*P* < 0.001, Tukey) were significantly higher than the low-grooming colonies. Similarly, the mean PDM was significantly higher in the selected high-grooming colonies (based upon DMMR) than in selected low-grooming colonies (*F*_1,20_ = 6.74, *P* = 0.017; Fig. 2A). When high-grooming colonies were categorized based on high and moderate damage, a significant effect of selection on damage was also found (*F*_2,19_ = 4.50, *P* = 0.025; Fig. 2B). The high-damage category showed significantly more damage than the low-grooming colonies (*P* = 0.019, Tukey). Meanwhile, the moderate-damage subgroup was intermediate and not significantly different from the low-grooming colonies (*P* = 0.59, Tukey) or from the high-damage subgroup (*P* = 0.37, Tukey).

### Intense Grooming

**Fig. 3.**
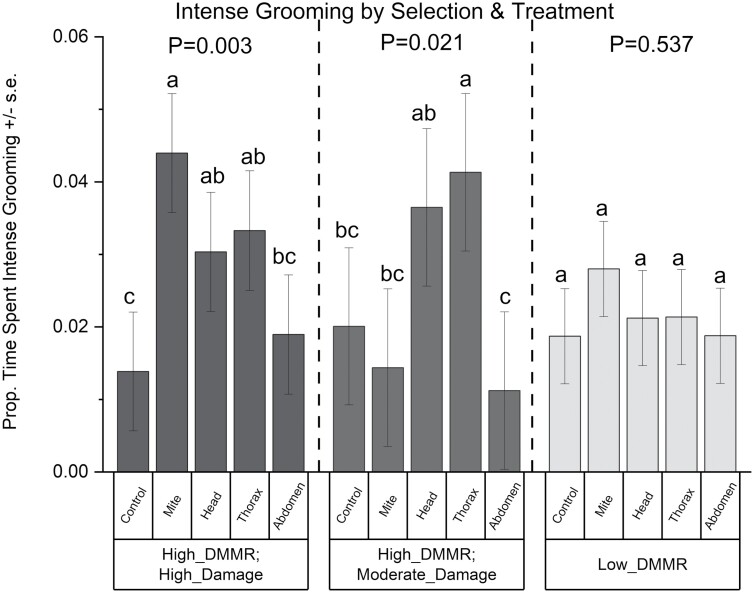
The proportion of time spent on intense grooming ± SE by selection and treatment for high-DMMR/high-damage colonies, high-DMMR/moderate-damage colonies, and low-DMMR colonies. A marginally significant selection-method*treatment interaction was found (see text), warranting further exploration. Slicing the data by selection method found a significant effect of treatment within the high-DMMR/high-damage and high-DMMR/moderate-damage subgroups, but not within the low-DMMR subgroup. Means represented by bars followed by the same letter within groups are not significantly different (*P* ≤ 0.05, protected LSD).

There was no overall effect of the selection method on the proportion of time that individual bees spent intensively grooming when comparing across all 5 treatment methods (*F*_2,19_ = 0.23, *P* = 0.79). However, the selection-method*treatment interaction was (*F*_8,75_ = 2.05, *P* = 0.052), warranting further exploration (Fig. 3) as recommended by Snedecor and Cochran (1989) for interactions between the 5% and 10% level when the value of *F* is greater than 1. Partitioning the interaction by treatment indicated that intense grooming was similar within all 5 treatment groups (control, mite application, and chalk dust applied to the head, thorax, or abdomen). However, partitioning the data by selection method showed that treatment effects varied significantly depending on the method used to select colonies. There was an effect of treatment on intense grooming within the high-DMMR/high-damage colonies (*F*_4,75_ = 4.40, *P* = 0.003, Slice) as well as within the high-DMMR/moderate-damage colonies (*F*_4,75_ = 3.07, *P* = 0.021, Slice), but not within the low-grooming colonies (*F*_4,75.2_ = 0.79, *P* = 0.537, Slice) (Fig. 3).

Within the high-DMMR/high-damage colonies, bees exposed to mites exhibited more intense grooming than control bees (*P* = 0.0004) or bees treated on the abdomen with chalk dust (*P* = 0.002). Bees treated with chalk dust on the head or thorax showed greater responses than the controls (*P* = 0.044 and *P* = 0.021, respectively).

Interestingly, within the high-DMMR/moderate-damage colonies, control bees showed less intense grooming than bees with chalk dust applied to the thorax (*P* = 0.050); however, bees exposed to mites did not groom more intensely than any other treatment, including controls (*P* > 0.05). Chalk dust treatment applied to the thorax elicited greater responses than mites (*P* = 0.019), while chalk dust applied to the head elicited only marginally greater responses than mites (*P* = 0.051). Applications of chalk dust to the head and thorax both showed significantly higher levels of intense grooming than chalk dust applied to the abdomen (*P* = 0.020 and *P* = 0.007, respectively). The exposure of chalk dust on the head showed a trend toward more intense grooming than the controls but was not significant (*P* = 0.121).

### Mite Removal

Successful mite removal was extremely rare in our assay, occurring only 9 times out of a total of 550 mite additions, 4 of which were removed by high-grooming bees and 5 by low-grooming bees.

## Discussion

This study analyzed the grooming responses of individual bees from colonies that were previously chosen based on high- and low-grooming behavior using mite drop and mite damage as indicators of grooming from broodless units. When studying grooming at the individual level, many metrics are used to quantify grooming including the proportion of time spent intensively grooming as used in our study and other measures such as the time to initiate grooming (e.g., Guzman-Novoa et al. 2012, Rivera-Marchand et al. 2012, Invernizzi et al. 2015, Dadoun et al. 2020, Morfin et al. 2020a, 2023, Russo et al. 2022). We quantified the proportion of time spent on intense grooming, which should be comparable to other studies that focused on the number of intense grooming responses or the number of bees performing intense grooming (Guzman-Novoa et al. 2012, Russo et al. 2022). Our approach was designed to precisely time the behavior using a grooming timer, which is an alternative metric to quantify grooming behavior. Using this metric, we show that bees respond to stimulus from *Varroa* more than from a standardized long-lasting stimulus under some treatment combinations and that bees selected using different criteria can respond more to stimuli in different body regions, but these responses depend upon the grooming ability of their parent source colony. While bees in the low-grooming group of colonies showed no significant differences among treatments of either type of stimulus applied (mites or chalk dust) versus control bees (which suggests a lack of ability to discriminate between mites and other irritants), significant differences among stimuli were found in both high-grooming categories. Colonies that were chosen based on high mite mortality rates *and* high mite damage rates demonstrated an ability to discriminate *Varroa* more than a standard stimulus. Most notably, individuals from high-grooming colonies responded with longer bouts of intense grooming when a stimulus was applied to the head or thorax, compared to sham-stimulated controls, while bees from low-grooming colonies showed no differences between stimulated and sham-stimulated bees.

Developing molecular and proteomic markers for use in queen breeding programs for a variety of traits associated with better hygiene in brood and adult honey bees shows great promise (Lapidge et al. 2002, Arechavaleta-Velasco et al. 2012, Tsuruda et al. 2012, Boutin et al. 2015, Guarna et al. 2017, Hamiduzzaman et al. 2017, Scannapieco et al. 2017, Morfin et al. 2020b, 2023), but a good understanding of specific behaviors and/or mechanisms involved in resistance against *Varroa* will be required as multiple genes are likely involved. Arechavaleta-Velasco et al. (2012), for example, identified over 27 candidate genes correlated with grooming behavior, including 3 genes associated with neurodevelopmental and behavioral effects. One gene, Neurexin-1 (AmNrx-1), is known to be associated with self-grooming behavior in mice (Arechavaleta-Velasco et al. 2012), intense grooming in honey bees (Hamiduzzaman et al. 2017), and mutilation of *Varroa* mites through biting responses (Morfin et al. 2020b). Other genes have been correlated with intense and light grooming (pUF68), and another with the presence of a mite on a bee (CYP9Q3) (Hamiduzzaman et al. 2017).

Although our study did not look into genetic markers, the pattern of heightened intense grooming in our high-damage subgroup after a *Varroa* stimulus also suggests there are differences in how bees from colonies with high mite-biting behavior react to stimuli. Importantly, the fact that these differences were only apparent in our high-damage subgroup suggests that alternate stimuli, like the chalk dust we used or wheat flour (as in Morfin et al. 2020a), may not be as effective at screening for bees that have all the capabilities needed for successful grooming of *Varroa.*Morfin et al. (2020b) collected random samples of bees from selected “mite-biter” and unselected Italian stock and did not run grooming assays prior to genomic analysis, which, as the authors suggest themselves, may have led to stronger correlations with AmNrx-1 had they done so. A subsequent study by Morfin et al. (2023), however, did use grooming assays to separate bees into subcategories of grooming ability prior to marker analysis—discovering 4 odorant-binding proteins and a gustatory receptor that were identified as differentially expressed genes in colonies with low *Varroa* growth (LVG) and high *Varroa* growth (HVG). However, the study relied on wheat flour as the stimulus to assess grooming parameters in their assays, and the authors also point to issues differentiating between LVG and HVG groups using wheat flour to elicit intense grooming responses. Thus, it is unknown if results would have been different if *Varroa* had been used as the stimulus. It is our belief that more specific attention to how bees identify and respond to cues produced by mites—as well as other behavioral traits that allow recruitment of allo-groomers and effective communication of the presence of mites—should be considered when trying to correlate the presence of putative markers with grooming success.

Differences in the sensitivity of different body regions were found within both subgroups of high-grooming colonies in our study but again varied depending on the colony-level selection metrics used to establish each category. Other insects vary in sensitivity to dust in different body regions. Fruit flies (*Drosophila*), for example, respond to dust by grooming and do so in a specific sequence, cleaning from the anterior to posterior of their bodies (Zhang et al. 2020). The response in these flies is regulated by comparing ratios of stimulation between mechanical sensory inputs in the different body regions. We did not perform this type of analysis on bees in our assays, nor do we know if there is a directional sequence to grooming based on our data but this work should be done in the future. Given the way in which bees are adapted to groom pollen from their heads and bodies to store in their corbiculum (Jander 1976), the sequence and sensory mechanism may differ from *Drosophila* which is presumably only focused on the removal of an irritant. When stimulated with *Varroa* or chalk dust applied to the head or thorax in our experiment, bees from the high-DMMR/high-damage colonies were groomed intensively for a significantly longer proportion of time compared to the unstimulated control bees. A similar significant difference was observed within the high-DMMR/moderate-damage colonies when chalk dust was applied to the thorax compared to the control bees. Thus, the hypothesis that high-grooming bees would show heightened sensitivity to both *Varroa* as well as a secondary stimulus was supported within both selection methods, and significant differences among treatment stimuli were not observed within low-grooming selections. This finding corroborates the recent work of Morfin et al. (2020a), who used an alternate irritant of wheat flour to produce grooming responses (using much higher amounts of the stimulus at 20 mg of flour as compared with our 0.3 mg of chalk dust), as well as older work by Land and Seeley (2004) who used a puff chalk dust applied to the wing base (a different area of the thorax than we used). In their grooming assays, Russo et al. (2022) used a short touch with a paintbrush (which we also used as our “sham” control) and compared it to *Varroa* and saw a greater response to *Varroa* as did we in our high-DMMR/high-damage colonies. Given that our high-DMMR/high-damage colonies exhibited heightened *Varroa*-specific responses relative to the moderate-damage cohort, and in combination with other research described above, it is our belief that the decision to use alternate stimuli in grooming assays may not capture all of the factors that affect grooming success, and whenever possible, using *Varroa* mites in grooming assays should be given serious consideration.

An overall trend in both the high-damage and moderate-damage categories was less intense grooming responses when chalk dust was applied to the abdomen versus other regions. While this trend was not significantly lower than chalk applied to the other 2 body regions in the high-damage colonies, chalk applied to the abdomen resulted in lower grooming responses than when applied to the head and thorax in the moderate-damage colonies. These findings support our second hypothesis that high-grooming bees would exhibit differential sensitivity over different body regions. Ramsey et al. (2019) showed that *Varroa* has a strong preference for the ventrolateral abdomen, commonly found wedged between the sternites on the third metasomal segment, and with a significant preference for the left side. The authors point out that the preference for the abdominal region is likely due to the larger deposit of fat body tissue but also posit that the preference for the third segment may be because the mite is largely shielded from grooming by the host bee in this location. Our results show that the abdomen is also a location where mites may be less detectable by workers who would be less likely to activate grooming, although it should be noted we did not apply dust to the underside of the abdomen where *Varroa* commonly resides. The fact that there was a significant effect of body region in the moderate-damage colonies but less evident in the high-damage colonies could be partly because the level of intense grooming when dust was applied to the abdomen was also slightly higher in the high-damage colonies, thus reducing the relative difference over body regions relative to the moderate-damage group. As some other research also suggests (Russo et al. 2022), high-DMMR/high-damage bees may have heightened overall sensitivity to foreign stimuli, and bees selected based upon more than one criterion may have a better capacity to recognize and respond to mites through grooming. This hypothesis needs to be confirmed with more detailed work on mechanoreceptor physiology to confirm it. Since *Varroa* are more commonly found on the abdomen, the high-DMMR/high-damage bees may also have heightened sensitivity to any stimulus on the abdominal region and may initiate grooming responses at a lower threshold of stimulation. However, this difference, if it occurs was not large enough to be detected within our study, perhaps due to the lower sample size in our moderate-damage category.

Successful mite removal was observed only a small number of times in our assays. Previous studies by Guzman-Novoa et al. (2012), Hamiduzzaman et al. (2017), and Dadoun et al. (2020) have all found intense grooming behavior to be associated with increased removal of mites in Petri dish assays. In the Dadoun et al. (2020) study, mite removal was particularly high, occurring in 60% of 240 trials involving resistant bees. Mite removal in the Guzman-Novoa et al. (2012) study was also high, ranging from 15% to 34.9% in bees of Africanized and Russian stocks—both of which are known to have high levels of mite-resistant traits. Moreover, their colonies were specifically selected for mite resistance over multiple years. Our study, however, used a diverse set of colonies made up mainly of beekeepers who used locally adapted stock but did not actively breed bees for grooming behavior or other *Varroa*-resistance mechanisms. Thus, it is possible that if we had used colonies selected for grooming behavior over multiple generations, there may have been larger differences between selected groups. Age can also affect grooming responses, with nurse-aged bees engaging more in grooming than bees greater than 14 days old (Dadoun et al. 2020, Russo et al. 2022), but it is not likely responsible for the lower grooming rates we observed. Although we did not paint mark our bees to avoid interference with grooming responses, we did select nurse bees from the brood nest, which should have been among the most responsive age group of bees.

The high-damage group cutoff rate of more than 30% of the mites being damaged that we used is in line with other studies that have measured mite damage. Guzman-Novoa et al. (2012), for example, found mite damage rates of between 26.2% and 59.5% in colonies with presumed mite resistance and rates of 7% and 23.8% in presumed mite-susceptible colonies. Andino and Hunt (2011) found mite damage rates of 7%–44% in their studies, which correlated the level of damage found in source colonies with the overall mite mortality rate in caged bees. The resistant colonies in Russo et al. (2022) had a mean damage rate of 44.2%. Hunt et al. (2016) also bred specifically for mite damage over many years, starting with a damage rate of about 3% and selectively breeding up to a damage rate of almost 50% 8 years later. However, none of these mite damage rates come close to the 73.8% damage rate noted in the study by Peng (1987) using *A. cerana.* Thus, although *A. cerana* is clearly a better groomer than *A. mellifera*, these studies do provide strong evidence that grooming behavior may be improved over time with selection.

A potential limitation of this study is that it is possible that the CO_2_ that was used to gently anesthetize the bees before placing them into the Petri dishes could have influenced their behavior. This was a trade-off done to slow the bees’ activity due to the challenges associated with applying a stimulus to a bee moving around in a Petri dish—particularly one vibrating its wings, causing the mite or chalk to blow around before successfully applying it to the bee. The anesthetization of the bees with CO_2_ was, in most cases, just enough to get the bees into the dishes and apply the stimuli. By then, the bees were usually awake and just beginning to move about in the dish, and after a few seconds of moving around the bees appeared to be back to “normal.” On rare occasions, marginally active bees were not noticed until they were watched more closely during video analysis. If bees were deemed to be unrecovered from the CO_2_ during video analysis, they were noted as such and were removed from the data set for any statistical analyses. It is also possible, however, that the CO_2_ influenced the bees’ ability to groom intensely or may have caused them to begin grooming earlier. On occasion, it was noted that bees that were returning to a normal level of activity began light grooming almost immediately (usually slowly cleaning their antennae) and continued to lightly groom after returning to a “normal” level of activity. However, since all bees in all stimulus treatments (including the controls) were treated the same, the relative differences we documented among treatments should be valid. Another possible limitation of this study is that there were multiple observers who assessed the grooming responses of bees in the videos. Thus, some subjectivity inevitably exists within the data. However, the care taken to train and calibrate observations, using recorded video that allowed each observer to watch the bees’ responses over again if needed, and the use of the grooming timer “app” all served to reduce subjectivity and made data collection much less subjective than other methods.

Overall, the results of this study help to clarify how individual bees identify and respond to the presence of general irritants versus *Varroa* through grooming responses. Our results reveal that high-grooming bees had heightened sensitivity to both *Varroa* and a stimulus of chalk dust compared to low-grooming bees. In addition, the pattern of heightened intense grooming in the *Varroa*-stimulated bees from high-damage colonies versus those from moderate-damage colonies suggests that *Varroa*-specific sensitivity is in some way linked with high mite damage rates. Thus, using alternative irritants such as chalk dust or wheat flour, while valuable in scoring generalized grooming responses, may not be able to select bees with all the components needed for the most successful grooming of *Varroa* at the colony level. Our results suggest that colonies with high damage rates, in addition to high mite mortality rates, may have additional sensory abilities, such as heightened tactile or chemosensory abilities, that allow them to more accurately target *Varroa*. In addition, auto-grooming is only part of a range of behaviors existing at the colony level, and it could be that allo-grooming, as well as communication signals used to recruit allo-groomers, also play a role in the successful grooming and injuring of mites in high-grooming colonies. Further work should be done to assess the correlations between colony level metrics (particularly mite damage), intense grooming responses in Petri dish assays using *Varroa* as the stimulus, allo-grooming and communication responses, and molecular and proteomic markers.
